# Physiological Tissue Response to Pulsed-Field Ablation for Atrial Fibrillation: Insights from Biomarker Kinetics

**DOI:** 10.33549/physiolres.935802

**Published:** 2026-08-01

**Authors:** Karol ZDRAVECKÝ, Jaroslav ZEMBJAK, Jan FARKAŠ, Silvia MIŠÍKOVÁ

**Affiliations:** 1 Department of Arrhythmology, Clinic of Cardiology and Angiology, Kardiocentrum AGEL, Košice-Šaca, Slovakia

**Keywords:** Pulsed-field ablation, Atrial fibrillation, Myocardial injury, Biomarkers

## Abstract

Pulsed-field ablation (PFA) has become an important non-thermal modality for pulmonary vein isolation in atrial fibrillation (AF), characterized by myocardial selectivity and reduced collateral tissue injury. However, the physiological biomarker response to PFA remains incompletely defined. This study assessed serial changes in myocardial (troponin T, creatine kinase - CK and its isoenzyme - CK-MB) and renal (urea, creatinine) biomarkers in patients undergoing PFA at a single center and compared these findings with current clinical evidence. The study cohort consisted of 52 patients undergoing PFA (73 % men, 27 % women), with 52 % presenting paroxysmal AF and 48 % persistent AF. The mean left atrial diameter was 45 ± 7 mm and the average left ventricular ejection fraction was 55 ± 7 % The mean ablation burden was 67 ± 23 applications per procedure. Biomarkers were measured before and after ablation. Repeated-measures ANOVA demonstrated significant time effects for all myocardial biomarkers (p < 0.001) and significant interactions with ablation burden. Troponin-T increased from near-zero baseline values to markedly elevated levels immediately and 24-hours after the procedure, while CK and CK-MB rose several-fold with only partial decline after the procedure. In contrast, urea and creatinine remained stable, indicating preserved renal function without acute kidney injury. The number of PFA applications correlated positively with late CK and CK-MB values, suggesting that myocardial biomarker load scales with procedural intensity. These findings align with current literature showing extensive myocardial biorelease after PFA but favorable extracardiac safety. By concurrently analyzing myocardial and renal markers, this study demonstrates that substantial biochemical myocardial injury induced by PFA does not translate into measurable renal dysfunction. Overall, PFA appears physiologically consistent with a profile of robust but targeted myocardial injury and preserved renal safety.

## Introduction

Catheter ablation of atrial fibrillation represents a cornerstone of rhythm-control therapy in symptomatic patients, with pulmonary vein isolation (PVI) constituting the primary procedural target. Conventional thermal ablation modalities, including radiofrequency (RF) and cryoballoon ablation, are effective but inherently limited by collateral tissue injury related to heat or freezing, affecting extracardiac structures such as the esophagus, phrenic nerve, and pulmonary veins. In addition, RF and cryoballoon ablation differ in procedural characteristics: cryoballoon ablation is generally associated with standardized procedure times, whereas RF ablation requires meticulous lesion delivery and validation, translating into greater operator dependency, with outcomes closely linked to catheter stability, lesion contiguity, and mapping expertise These limitations have driven the development of non-thermal ablation technologies aimed at improving procedural safety without compromising efficacy [1–3,17, 23].

Pulsed-field ablation is a novel non-thermal ablation modality based on irreversible electroporation (IRE), characterized by preferential susceptibility of cardiomyocytes and relative preservation of the extracellular matrix and adjacent non-cardiac tissues. However, PFA should not be considered a single uniform technology, as currently available systems differ substantially in generator design, waveform characteristics, catheter architecture, and biological effects. The present study specifically evaluates the Farapulse™ system (Boston Scientific), a biphasic bipolar pentaspline platform widely used for pulmonary vein isolation. Preclinical investigations have consistently demonstrated sharply demarcated myocardial lesions with minimal collateral injury to surrounding structures, including the esophagus, phrenic nerve, and pulmonary veins. These findings have been corroborated by early clinical trials and large multi-center registries, which reported a favorable extracardiac safety profile and rapid procedural efficiency, leading to the widespread adoption of PFA for atrial fibrillation ablation in routine clinical practice [1–3,17].

From a biophysical and electrophysiological perspective, PFA achieves tissue ablation through the delivery of high-voltage, ultrashort electrical pulses that induce a rapid increase in transmembrane potential, resulting in the formation of nanoscale aqueous pores within the lipid bilayer. When pulse parameters remain below a critical threshold, pore formation is transient and membrane integrity is restored (reversible electroporation); however, exceeding this threshold leads to persistent membrane destabilization, loss of ionic homeostasis, and irreversible cellular injury (IRE) [24–26]. At the cellular level, irreversible electroporation induces loss of membrane integrity, ionic imbalance, calcium overload, mitochondrial dysfunction, and subsequent cardiomyocyte death with predominantly necrotic or mixed regulated injury features rather than classical apoptosis alone. [26,27].

Importantly, the therapeutic window of PFA arises from tissue-specific electroporation thresholds, which differ across cell types and microarchitectural environments. Atrial cardiomyocytes demonstrate a lower susceptibility threshold to irreversible electroporation compared with several extracardiac tissues, enabling effective myocardial ablation while preserving neighboring structures [25,28,29]. Experimental human-cell models have further confirmed a greater vulnerability of cardiomyocytes to pulsed electric fields compared with esophageal epithelial cells under comparable conditions, providing a mechanistic explanation for the observed myocardial selectivity of PFA in clinical practice [29,30].

Despite this tissue selectivity, PFA induces extensive myocardial injury at the cellular level. Multiple clinical studies have consistently reported markedly higher post-procedural myocardial biomarker release, particularly troponin and creatine kinase, following PFA compared with conventional thermal ablation modalities [6–8,10]. These observations indicate that myocardial selectivity does not necessarily translate into smaller lesion volume, but rather reflects reduced off-target tissue injury. However, the physiological interpretation of this pronounced biomarker response - especially in relation to myocardial injury burden, lesion maturation, and extracardiac organ tolerance - remains incompletely defined.

While selected reports have described intravascular hemolysis and transient renal dysfunction following PFA - most commonly in patients with extensive ablation burden or impaired baseline renal function - data simultaneously evaluating myocardial and renal biomarker responses under standard procedural conditions are limited. In particular, studies integrating myocardial injury markers with renal function parameters in real-world PFA populations remain scarce [13–16].

We therefore aimed to characterize the time-dependent myocardial and renal biomarker response following pulsed-field ablation for atrial fibrillation. We hypothesized that PFA induces a robust myocardial biomarker release reflecting effective electroporation-mediated lesion formation without concomitant deterioration of renal function.

## Methods

### Study design and patient population

This prospective, single-center observational study included consecutive patients with atrial fibrillation undergoing first-time pulsed-field ablation (PFA) for ablation of atrial fibrillation at the Department of Arrhythmology, Clinic of Cardiology and Angiology, Kardiocentrum AGEL Košice–Šaca. Patients with paroxysmal or persistent atrial fibrillation referred for rhythm-control catheter ablation were eligible for inclusion.

Exclusion criteria comprised advanced chronic kidney disease (estimated glomerular filtration rate < 30 mL/min/1.73 m^2^) - such patients were not considered candidates for pulsed-field ablation at our institution during the study period and therefore were neither treated with PFA nor enrolled in the present analysis. Furthermore patients with acute infection or systemic inflammatory disease, recent myocardial infarction within the preceding three months, and incomplete biomarker data were also excluded from the study. We carefully selected patients undergoing pulsed-field ablation and all patients included signed informed consent about study participation.

### Pulsed-field ablation procedure

All procedures were performed under general anesthesia using a commercially available pulsed-field ablation system (Farapulse™, Boston Scientific) with the Farawave™ pentaspline catheter. Vascular access was obtained under ultrasound guidance with two separate punctures of the right femoral vein. An 11-French sheath was introduced for intracardiac echocardiography (ICE), and a transseptal sheath was placed for left atrial access.

The ICE catheter was advanced into the right atrium to guide all subsequent steps. A guidewire was positioned in the superior vena cava, and transseptal puncture was performed under continuous ICE visualization using a Brockenbrough (BRK) needle. Immediately after successful transseptal access and prior to energy delivery, 1mg of intravenous atropine was administered to mitigate vagal responses associated with pulsed-field energy application. A guidewire was then advanced into the left superior pulmonary vein, followed by exchange for the dedicated Faradrive™ steerable sheath.

Systemic anticoagulation was initiated immediately after left atrial access with intravenous unfractionated heparin, with repeated measurements of activated clotting time (ACT) to achieve and maintain a target ACT of >300 s throughout the procedure.

Pulmonary vein isolation was performed using the Farawave™ catheter according to the manufacturer-recommended workflow. For each pulmonary vein, energy delivery consisted of four applications in the basket configuration followed by four applications in the flower configuration, with systematic catheter rotations to ensure circumferential lesion formation. Additional targeted antral applications, most commonly in the flower configuration, were delivered when residual electrograms or incomplete electrical isolation were identified. Posterior wall isolation was performed in patients with persistent atrial fibrillation, no routine cavotricuspid isthmus, mitral isthmus, or other extra-pulmonary vein linear lesion sets were performed.

Pulse-field delivery parameters, including pulse amplitude, number, and waveform characteristics, were predefined by the system and applied uniformly across all procedures. Ablation burden was quantified as the total number of pulsed-field applications delivered per procedure.

Intracardiac echocardiography was used at the operator’s discretion to assist with catheter positioning and anatomical assessment of the left atrium.

### Periprocedural hydration protocol

To minimize hemodynamic variability and reduce the risk of procedure-related hemolysis and renal stress, a standardized intravenous hydration protocol was applied in all patients. This consisted of 250 mL of isotonic saline administered prior to transfer to the electrophysiology laboratory, 500 mL during the procedure, and an additional 500 mL following completion of the ablation.

### Biomarker assessment

Venous blood samples were obtained at three predefined time points: before the procedure (baseline), on the day of the procedure after completion of ablation, and 24 hours after the procedure. Myocardial injury was assessed using serum concentrations of cardiac troponin T, creatine kinase (CK), and CK-MB. Renal function was evaluated using serum urea and creatinine levels. All laboratory analyses were performed using standardized assays in the hospital’s certified clinical laboratory.

### Statistical analysis

Continuous variables were assessed for normality and are presented as mean ± standard deviation. Changes in myocardial and renal biomarkers across the three time points were analyzed using repeated-measures analysis of variance (RM-ANOVA). The assumption of sphericity was evaluated using Mauchly’s test; when violated, Greenhouse–Geisser correction was applied.

Post-hoc pairwise comparisons were performed with Bonferroni adjustment for multiple testing. Effect sizes were expressed as partial eta squared (η^2^) or Cohen’s d, as appropriate. Associations between ablation burden, defined as the total number of pulsed-field applications, and biomarker levels were assessed using correlation analysis.

A two-sided p value < 0.05 was considered statistically significant. Statistical analyses were performed using JASP statistical software.

## Results

### Patient characteristics

The study cohort comprised 52 patients (73 % men), with paroxysmal atrial fibrillation present in 52 % and persistent atrial fibrillation in 48 %. Mean left atrial diameter was 45 ± 7 mm and mean left ventricular ejection fraction was 55 ± 7 %. The mean ablation burden was 67 ± 23 (min. 28, max. 123) pulsed-field applications per procedure. Baseline renal function was preserved in all patients. Baseline characteristics of the study population are summarized in Table 1. The cohort consisted predominantly of middle-aged men with preserved left ventricular function and a high prevalence of arterial hypertension. No patient had advanced chronic kidney disease at baseline.

### Myocardial biomarker response

Repeated-measures ANOVA demonstrated a significant time-dependent effect for all myocardial biomarkers. For creatine kinase (CK), a significant main effect of time was observed (Greenhouse–Geisser corrected F(1.36, 55.57) = 4.47, p = 0.028, η^2^ = 0.026), with mean creatine kinase increased from 2.21 ± 1.60 μkat/L at baseline to 10.58 ± 4.61 μkat/L immediately after the procedure and remaining elevated at 8.95 ± 4.47 μkat/L at 24 hours. Post-hoc analysis confirmed significant differences between baseline and after the procedure as well as baseline and 24-hour values (both p < 0.001). Raincloud plots demonstrate a marked upward shift and increased dispersion of CK values during the procedure, followed by partial normalization at 24 hours (Fig. 1A).

Similarly, CK-MB showed a significant effect of time (F(1.39, 63.95) = 3.75, p = 0.044, η^2^ = 0.026), increasing from 0.32 ± 0.23 μg/L at baseline to 1.45 ± 0.61 μg/L during the procedure and remaining elevated at 0.83 ± 0.35 μg/L at 24 hours (all post-hoc comparisons vs. baseline p < 0.001).

Raincloud visualization illustrates a broadening of the CK-MB distribution at intraprocedural and 24-hour measurements, indicating interindividual variability in myocardial enzyme release (Fig. 1B). Cardiac troponin T exhibited a pronounced time-dependent increase (F(1.29, 55.51) = 5.42, p = 0.016, η^2^ = 0.039), rising from near-baseline concentrations (0.010 ± 0.007 μg/L) to 1.63 ± 0.82 μg/L after the procedure and 1.65 ± 0.80 μg/L at 24 hours. No significant difference was observed between intraprocedural and 24-hour troponin T levels (p = 1.000), indicating an early plateau of troponin release following pulsed-field ablation.

This kinetic pattern is reflected in the raincloud plot by a uniform upward displacement of the troponin T distribution with minimal change in dispersion between intraprocedural and 24-hour time points (Fig. 1C).[Fig f1d-pr75_667]

A significant interaction between time and ablation burden was observed for CK (F(1.36, 55.57) = 11.79, p < 0.001, η^2^ = 0.068), CK-MB (F(1.39, 63.95) = 8.90, p = 0.002, η^2^ = 0.061), and troponin T (F(1.29, 55.51) = 5.74, p = 0.014, η^2^ = 0.041), demonstrating that higher numbers of pulsed-field applications were associated with greater myocardial biomarker release.

### Renal biomarker response

In contrast to myocardial biomarkers, renal parameters remained stable throughout the peri-procedural period. Repeated-measures ANOVA revealed no significant time-dependent changes in serum urea (F(1.50, 67.51) = 1.60, p = 0.214, η^2^ = 0.007) or creatinine levels (F(1.51, 67.81) = 0.28, p = 0.696, η^2^ < 0.001).

Mean serum urea values showed minimal variation across time points (5.75 ± 1.35 mmol/L at baseline, 5.66 ± 1.22 mmol/L intraprocedurally, and 5.48 ± 1.29 mmol/L at 24 hours), while creatinine values remained within physiological variability (95.7 ± 15.6 μmol/L at baseline, 91.5 ± 16.1 μmol/L intraprocedurally, and 92.9 ± 17.6 μmol/L at 24 hours). Although a minor intraprocedural decrease in creatinine was detected on post-hoc analysis (p = 0.044), this change was transient and not clinically relevant.

Raincloud plots show substantial overlap and tight clustering of urea and creatinine distributions across all time points, supporting preserved renal function during the peri-procedural period (Fig. 2A–B). No patient fulfilled criteria for acute kidney injury during the observation period.

### Relationship between ablation burden and biomarker levels

Correlation analysis demonstrated moderate positive associations between ablation burden and myocardial biomarker levels. The total number of pulsed-field applications correlated significantly with late CK (r ≈ 0.46, p = 0.003) and CK-MB values (r ≈ 0.44, p = 0.001), supporting a dose–response relationship between procedural intensity and myocardial biochemical injury.

This association is visually reflected by the rightward shift and widening of CK and CK-MB distributions on raincloud plots at later time points (Fig. 1A–B).

In contrast, no significant correlations were observed between ablation burden and renal biomarkers, including urea and creatinine (all p > 0.30).

## Discussion

In this prospective single-centre cohort of patients undergoing pulsed-field ablation for atrial fibrillation, we observed a characteristic physiological response marked by pronounced, procedure-related myocardial biomarker release alongside preserved renal function throughout the peri-procedural period. Creatine kinase, CK-MB, and troponin T increased substantially following ablation, whereas urea and creatinine remained stable, and no patient fulfilled criteria for acute kidney injury. These findings provide integrated evidence that extensive biochemical myocardial injury induced by PFA does not necessarily translate into extracardiac organ dysfunction under routine clinical conditions. The relatively young mean age of our cohort (45.4 ± 6.7 years) should also be acknowledged when interpreting the findings. This likely reflects local referral patterns, earlier rhythm-control strategies in symptomatic atrial fibrillation patients, and selection of individuals considered suitable for catheter ablation. Consequently, extrapolation of the present renal safety observations to older and more comorbid populations, particularly those with impaired baseline renal function, should be made with caution.

### Myocardial biomarker kinetics and electroporation physiology

In our cohort, myocardial biomarker release followed a consistent, application-dependent kinetic profile. Creatine kinase (CK) and CK-MB rose sharply during the procedure, followed by a partial decline at 24 hours, whereas cardiac troponin T demonstrated a rapid early increase with subsequent plateau formation. This temporal dissociation suggests differences in biomarker biology and intracellular handling rather than divergent mechanisms of myocardial injury.

The overall magnitude of myocardial biomarker elevation observed in our study aligns with previous comparative analyses reporting higher post-procedural troponin and CK release after pulsed-field ablation compared with radiofrequency or cryoballoon ablation [11]. Importantly, these biochemical changes should not be interpreted as pathological or indicative of procedural complications. Instead, they reflect effective electroporation-mediated lesion formation, supporting the concept that PFA induces substantial myocardial injury at the cellular level while preserving tissue selectivity [10–13].

The significant interaction between time and ablation burden further reinforces this interpretation. Higher numbers of pulsed-field applications were associated with greater myocardial biomarker release, demonstrating a clear dose–response relationship between procedural intensity and biochemical injury. These findings underscore the importance of procedural precision and avoidance of unnecessary energy delivery, even within a non-thermal ablation paradigm [9,10,13].

From a physiological perspective, the divergent kinetic behavior of cardiac troponin T and creatine kinase–based biomarkers provides additional insight into the underlying injury phenotype induced by PFA. Troponin T is a structural myofibrillar protein tightly bound to the contractile apparatus and is released predominantly in association with cytoskeletal disruption and delayed necrotic or mixed injury processes, resulting in prolonged circulation time and plateau-type kinetics. [18–20]. In contrast, CK and CK-MB are cytosolic enzymes that rapidly diffuse into the circulation following membrane permeabilization and are cleared more quickly, leading to greater temporal variability and sensitivity to procedural intensity [8,10].

Irreversible electroporation is believed to induce cardiomyocyte injury primarily through disruption of membrane integrity, loss of cellular homeostasis, and subsequent cell death pathways with predominantly necrotic or mixed morphological features, rather than through classical apoptosis alone. This mechanism may help explain the substantial post-procedural release of intracellular myocardial biomarkers observed after pulsed-field ablation. This mechanistic profile offers a plausible explanation for the sustained troponin T plateau observed after PFA, alongside the more dynamic behavior of CK and CK-MB. Similar dissociations between troponin and creatine kinase kinetics have been consistently reported in prior clinical PFA studies across different catheter platforms, further supporting the notion that myocardial biomarker release after PFA reflects the specific injury phenotype rather than lesion inefficiency [10–13,18–21].

### Renal safety and extracardiac tolerance

In contrast to the robust myocardial response, renal biomarkers remained stable throughout the peri-procedural period. Neither urea nor creatinine exhibited clinically meaningful time-dependent changes, and no patient developed acute kidney injury. These renal findings should be interpreted within the context of the studied population. The absence of acute kidney injury likely reflects not only the procedural characteristics and hydration regimen, but also the exclusion of advanced chronic kidney disease and the relatively moderate application burden. Therefore, extrapolation to older, frailer, or renally impaired populations undergoing more extensive pulsed-field delivery should be made cautiously. [11,13].

Previous studies have identified intravascular hemolysis and transient renal impairment following PFA, predominantly in patients with reduced baseline renal reserve, hyperviscosity states, or extensive ablation burden [12,14]. In our cohort, preserved baseline renal function, controlled ablation protocols, and standardized periprocedural hydration may have contributed to the absence of measurable renal dysfunction. While minor intraprocedural fluctuations in creatinine were detected statistically, these changes were transient and lacked clinical relevance.

Importantly, the absence of renal deterioration despite pronounced myocardial biomarker release underscores that biochemical myocardial injury alone is not a reliable surrogate for extracardiac risk. This distinction is clinically relevant, as elevated troponin or creatine kinase values following PFA should be interpreted within a broader physiological and procedural context rather than prompting reflexive concern for systemic complications [8,10,11].

Although direct hemolysis markers such as plasma free hemoglobin, haptoglobin, or bilirubin were not systematically assessed, the preserved renal function observed in our cohort may indirectly suggest a low degree of clinically relevant intravascular hemolysis. This interpretation is consistent with recent reports indicating a dose-dependent relationship between pulsed-field application burden and hemolysis, with more pronounced biochemical hemolysis typically reported at substantially higher application counts than those used in the present study. In addition, the standardized periprocedural hydration protocol and sufficient tissue contact with catheter controlled by ICE may have further mitigated transient pigment-related renal stress.

### Comparison with published PFA safety data

Large registries and multicenter studies have consistently demonstrated a favorable extracardiac safety profile of pulsed-field ablation. The MANIFEST-17K registry reported extremely low rates of major complications, including the absence of confirmed atrio-esophageal fistula, across a broad spectrum of operators and procedural settings [2]. In contrast, the NEMESIS-PFA study identified hemolysis-related laboratory abnormalities and transient renal impairment primarily in selected high-risk subgroups, highlighting that extracardiac effects of PFA may be modulated by patient-specific and procedural factors [4].

Our findings align with these observations by demonstrating preserved renal function in a real-world cohort treated under standardized procedural conditions, thereby extending existing safety data through simultaneous assessment of myocardial and renal biomarker responses [11–13].

### Catheter technology, pulse-field delivery, and biomarker kinetics

Interpretation of myocardial biomarker kinetics after pulsed-field ablation must also take into account the specific catheter design and pulse-field delivery strategy. In the present study, pulmonary vein isolation was performed using the Farawave™ pentaspline catheter within the Farapulse system, which delivers bipolar, microsecond-scale, high-voltage pulsed electric fields in predefined configurations. Each pulmonary vein was treated using a standardized manufacturer-recommended protocol consisting of multiple applications in both flower and basket configurations, with systematic catheter rotations to achieve circumferential lesion formation. Although pulse amplitude, pulse number, and waveform characteristics are predefined by the system, this approach enables highly reproducible energy delivery while limiting operator-dependent variability [17,21,22].

From a biophysical perspective, the Farawave catheter geometry allows simultaneous engagement of a broad antral surface area, resulting in relatively homogeneous myocardial exposure to the electric field. This design may contribute to the pronounced and uniform troponin T release observed in our cohort, characterized by an early plateau pattern, while CK and CK-MB demonstrated greater temporal variability and sensitivity to ablation burden. Fixed-parameter, multi-electrode PFA systems such as Farapulse therefore prioritize lesion reproducibility over operator-adjustable pulse modulation, which may influence biomarker kinetics and hemolytic stress profiles [18–20,22].

Contemporary pulsed-field ablation platforms differ substantially in catheter architecture and pulse-field delivery philosophy. Systems employing expandable lattice or spherical electrode designs, as well as focal or semi-focal electrode arrangements, may generate more heterogeneous electric field distributions and allow varying degrees of pulse customization. Comparative experimental and early clinical data suggest that such differences can translate into variability in myocardial biomarker release, hemolysis, and renal stress markers, even when targeting similar anatomical regions [21–23]. These observations underscore that myocardial biomarker kinetics after PFA are influenced not only by lesion extent but also by field uniformity, electrode–tissue coupling, and pulse sequencing strategy.

Importantly, irrespective of system-specific differences, irreversible electroporation induces cardiomyocyte injury primarily through membrane destabilization, loss of cellular homeostasis, and subsequent necrotic or mixed regulated cell-death pathways rather than immediate thermal coagulative necrosis. This shared mechanistic substrate may explain the consistently higher troponin release observed across PFA technologies compared with thermal ablation, while allowing for variability in CK and CK-MB behaviour depending on catheter design and ablation burden [18–21].

Intracardiac echocardiography was used at the operator’s discretion to assist with catheter positioning and anatomical assessment of the left atrium. In the context of contemporary electrophysiological practice, meticulous catheter guidance and stable tissue contact—commonly supported by intracardiac imaging—together with standardized periprocedural hydration are regarded as key components for achieving reproducible lesion formation and minimizing unnecessary energy delivery and hemolysis-related stress [17,21].

### Clinical implications

These findings have several practical implications. First, pronounced myocardial biomarker elevation following PFA should be regarded as an expected physiological consequence of effective electroporation rather than a marker of procedural failure or ischemic injury. In the absence of supporting clinical or electrocardiographic evidence, elevated post-procedural troponin levels should not trigger unnecessary acute coronary syndrome workup. Second, renal safety appears favorable in patients without advanced pre-existing kidney disease when appropriate hydration and procedural catheter position control *via* intracardiac echocardiography are applied. Finally, integrated assessment of myocardial and renal biomarkers provides a more comprehensive evaluation of PFA safety than isolated biomarker measurements.

## Conclusions

In this prospective cohort, pulsed-field ablation was associated with marked myocardial biomarker release consistent with effective electroporation-mediated lesion formation, while short-term renal function remained preserved. No patient fulfilled KDIGO criteria for acute kidney injury under conditions of preserved baseline renal function, moderate application burden, and standardized hydration. These findings support pulsed-field ablation as a physiologically distinct non-thermal modality combining robust myocardial tissue effect with favorable short-term extracardiac tolerance.

Pulsed-field ablation induces a pronounced and expected rise in myocardial biomarkers, including CK, CK-MB, and troponin T, reflecting effective electroporation-mediated myocardial lesion formation. This biochemical response is consistent with the physiological mechanism of irreversible electroporation and should be interpreted as a marker of procedural myocardial injury rather than pathological ischemia.

Despite the substantial myocardial biomarker release, renal function remained stable throughout the peri-procedural period. The absence of measurable acute kidney injury, together with unchanged urea and creatinine levels, indicates preserved renal tolerance to PFA under routine clinical conditions in patients without advanced pre-existing kidney disease and individual-level analysis further showed that no patient fulfilled KDIGO criteria for acute kidney injury during the observed periprocedural period.

These findings should be interpreted in the context of contemporary electrophysiological practice, in which meticulous catheter guidance - commonly supported by intracardiac echocardiography - and standardized periprocedural hydration are regarded as key components for optimizing tissue contact and minimizing hemolysis-related stress.

Overall, these results support pulsed-field ablation as a physiologically distinct non-thermal ablation modality that combines robust myocardial lesion formation with favorable extracardiac safety, reinforcing its role in rhythm-control therapy for atrial fibrillation.

## Limitations

This study has several limitations. First, its single-center design and moderate sample size may limit generalizability. Second, follow-up was restricted to the acute post-procedural phase. Third, more sensitive markers of subclinical renal injury (e.g. neutrophil gelatinase-associated lipocalin, cystatin C) and hemolysis (e.g. free hemoglobin, haptoglobin) were not systematically measured. Finally, clinical outcomes such as symptomatic acute kidney injury, pericardial complications, or post-cardiac inflammatory syndromes were not primary endpoints of this analysis and patients with severe kidney disease were not the main focus of the study.

## Figures and Tables

**Fig. 1A):**
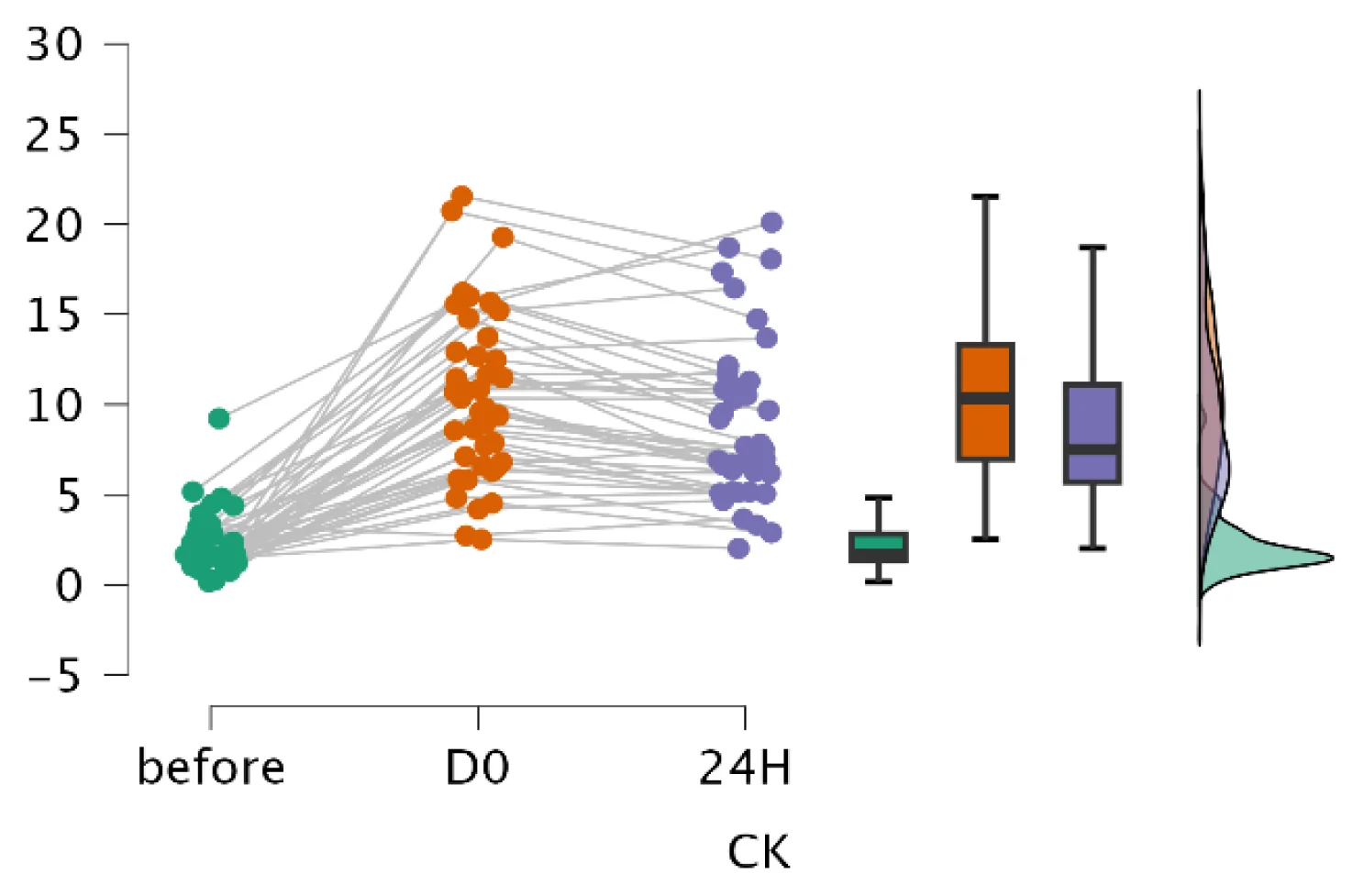
Changes in creatine kinase (CK) levels measured before the procedure (baseline), immediately after the procedure (D0), and 24 hours post-procedure (24H). Individual patient values are shown with paired connections illustrating within-subject changes over time. The accompanying boxplots and density plots summarize the distribution of CK levels at each time point, demonstrating a marked increase immediately after the procedure with partial decline at 24 hours, while remaining elevated compared with baseline.

**Fig. 1B):**
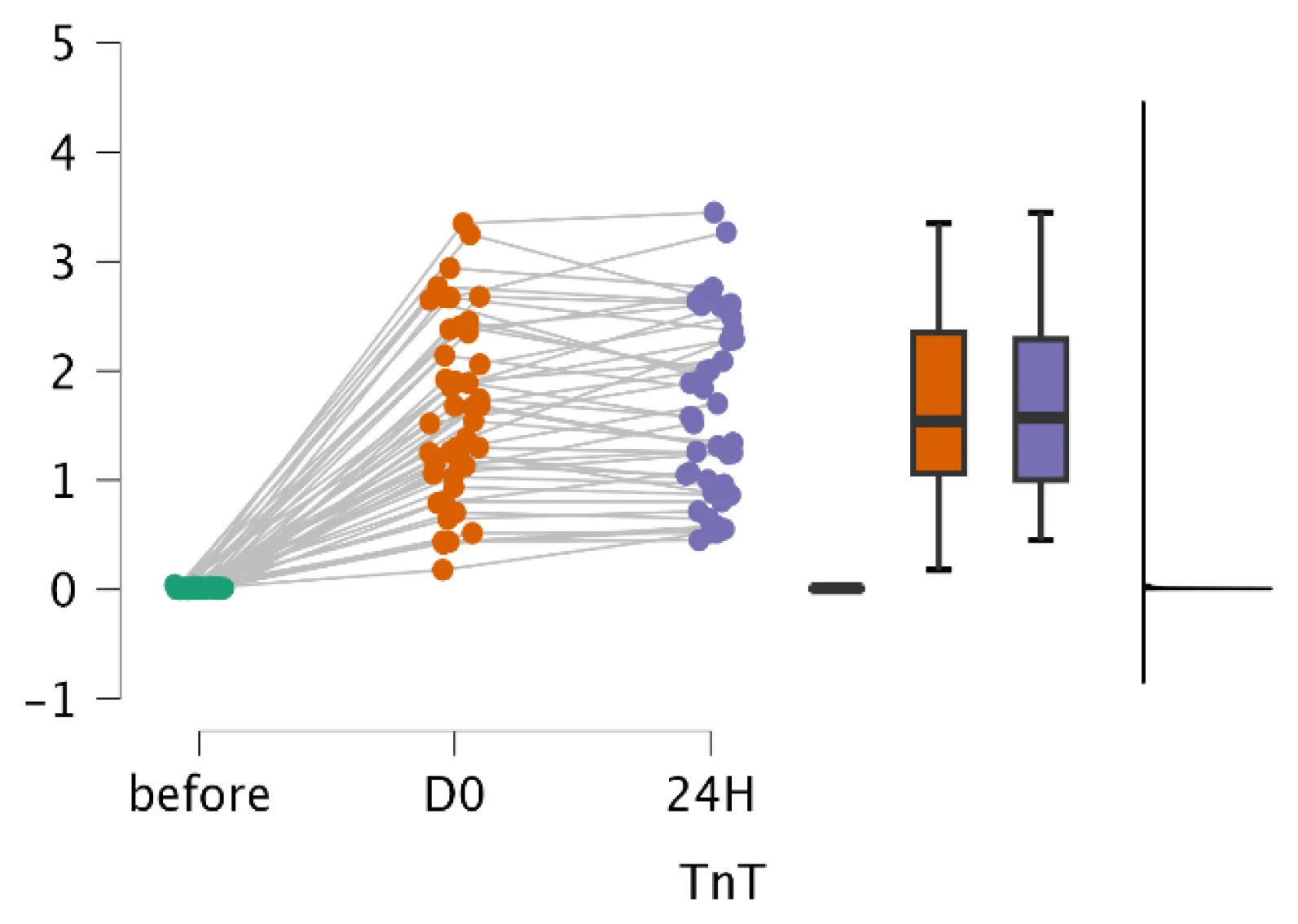
Changes in creatine kinase–MB (CK-MB) levels measured before the procedure (baseline), immediately after the procedure (D0), and 24 hours post-procedure (24H). Individual paired measurements illustrate within-subject dynamics. CK-MB levels show a clear periprocedural increase with partial normalization at 24 hours, as summarized by boxplots and distribution curves

**Fig. 1C):**
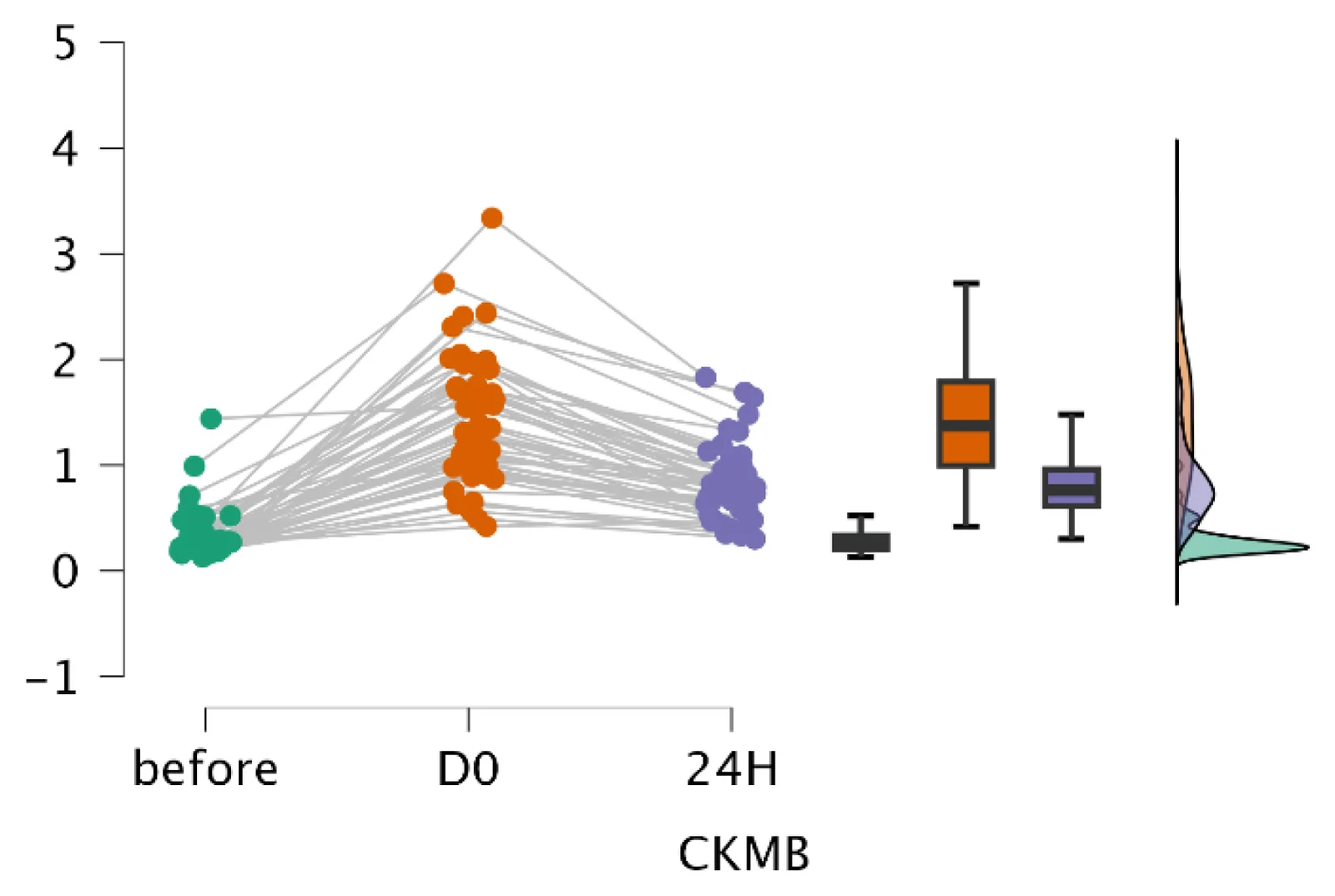
Temporal profile of high-sensitivity troponin T (TnT) concentrations assessed before the procedure (baseline), immediately after the procedure (D0), and 24 hours post-procedure (24H). Paired data demonstrate a pronounced acute post-procedural rise with sustained elevation at 24 hours.

**Fig. 1D) f1d-pr75_667:**
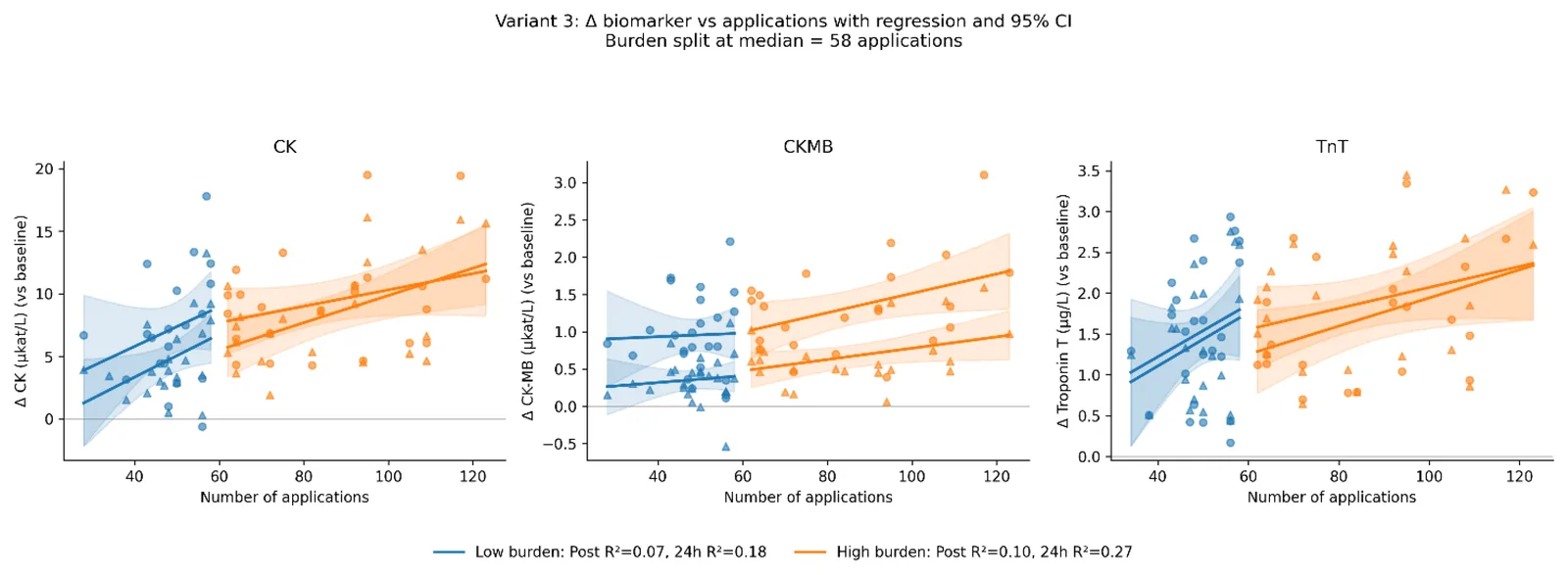
Scatter plots illustrate changes in creatine kinase, CK-MB, and troponin T relative to baseline at post-procedural and 24-hour time points according to ablation burden. Linear regression lines with 95 % confidence intervals demonstrate a positive association between the number of pulsed-field ablation applications and biomarker elevation; R^2^ values indicate the proportion of variance in biomarker change explained by ablation burden. These findings are consistent with a significant time × ablation burden interaction for CK (F(1.36, 55.57) = 11.79, p < 0.001, η^2^ = 0.068), CK-MB (F(1.39, 63.95) = 8.90, p = 0.002, η^2^ = 0.061), and troponin T (F(1.29, 55.51) = 5.74, p = 0.014, η^2^ = 0.041).

**Fig. 2A):**
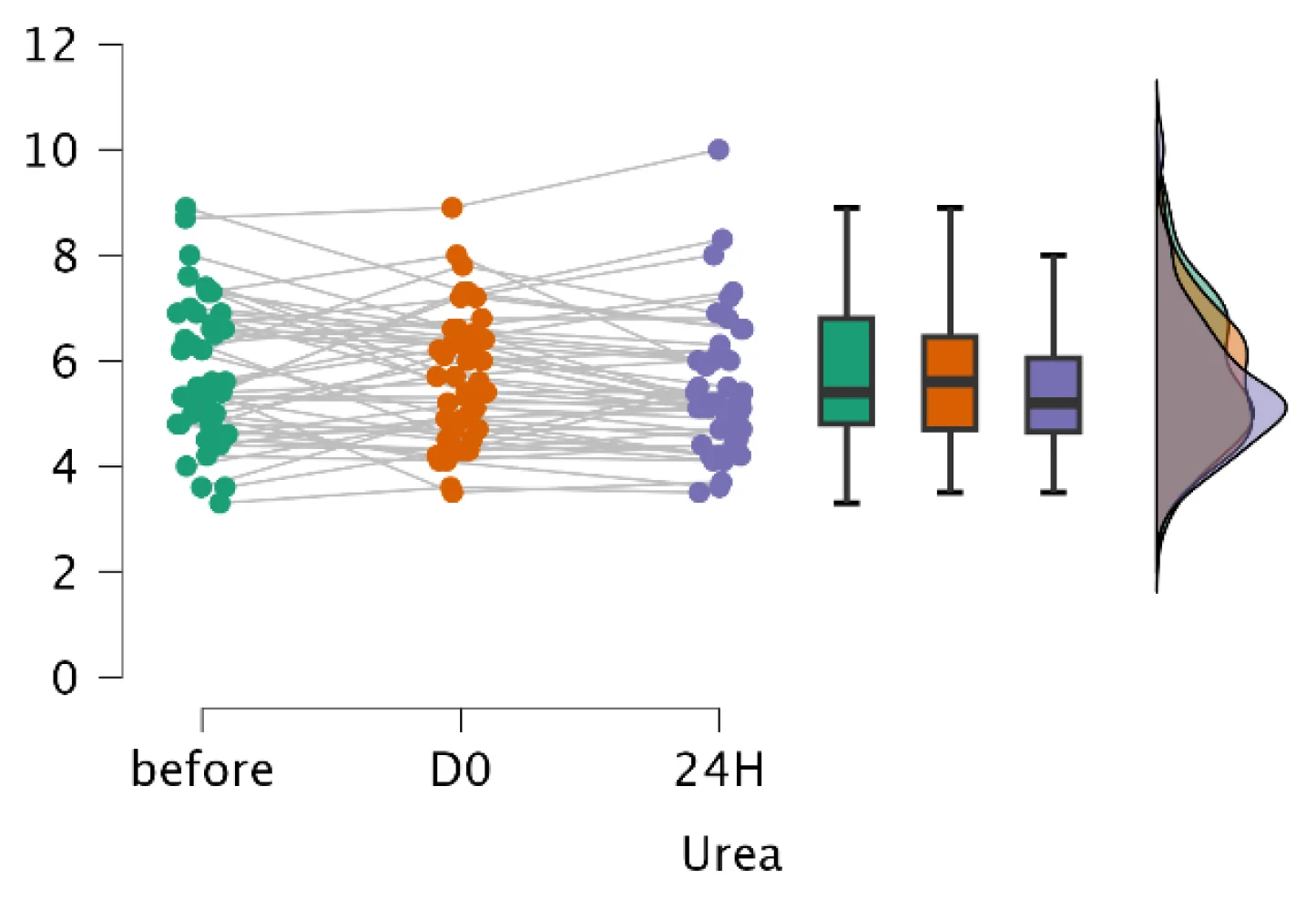
Temporal changes in serum urea levels measured before the procedure (baseline), immediately after the procedure (D0), and 24 hours post-procedure (24H). Individual paired measurements illustrate within-subject variability over time. Urea concentrations remain largely stable across all time points, with no evidence of clinically relevant periprocedural renal impairment.

**Fig. 2B):**
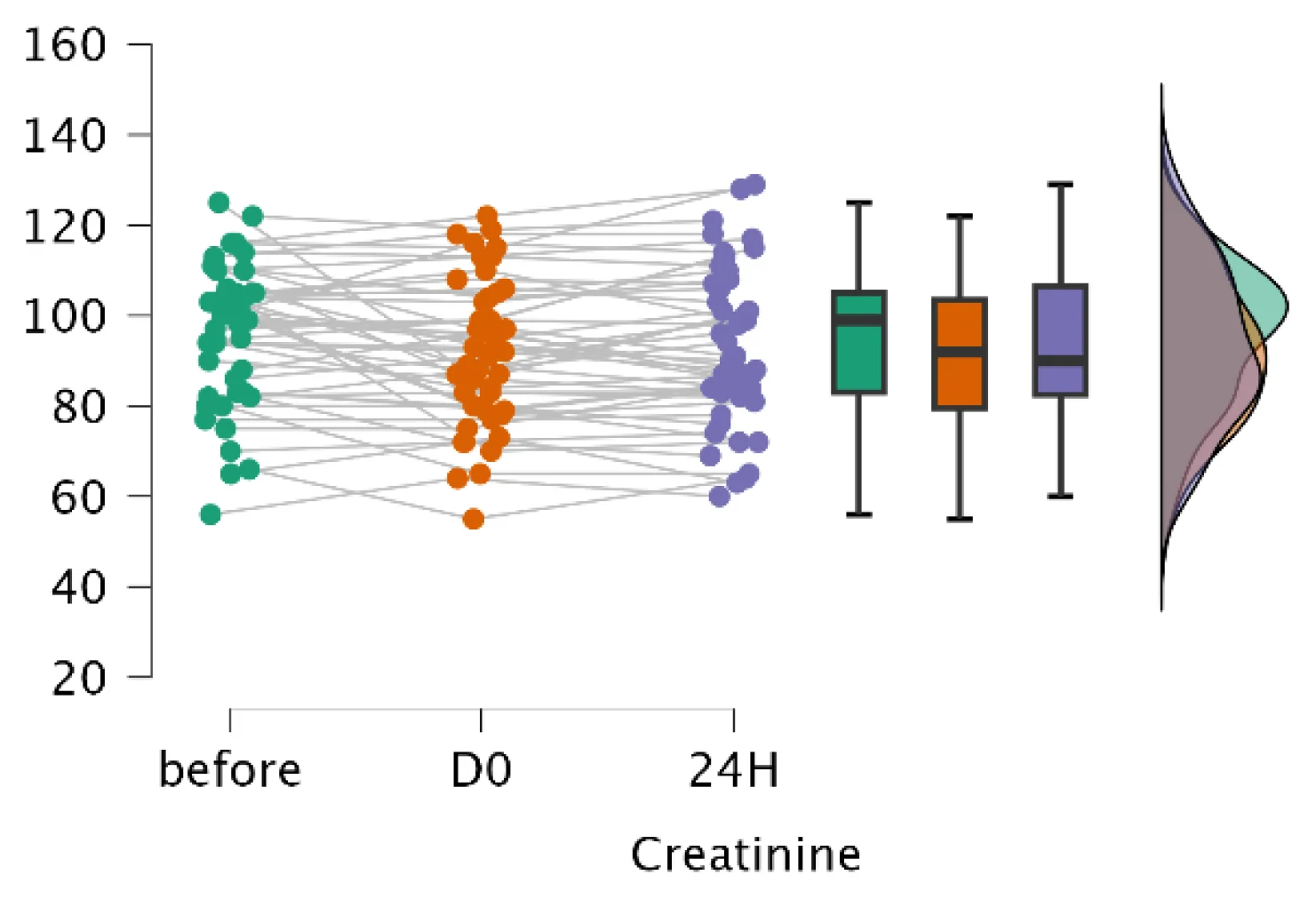
Changes in serum creatinine levels measured before the procedure (baseline), immediately after the procedure (D0), and 24 hours post-procedure (24H). Individual trajectories and summary distributions indicate relative stability of renal function across all time points, without clinically relevant periprocedural deterioration.

**Table 1:** Baseline characteristics of the study population

Characteristic	Total (n = 52)	Men (n = 38)	Women (n = 14)
Age, years	45.4 ± 6.7	46.5 ± 6.4	42.4 ± 6.8
Paroxysmal AF, n (%)	27 (52 %)	23 (60 %)	4 (15 %)
Persistent AF, n (%)	25 (48 %)	15 (40 %)	10 (85 %)
Left atrial diameter, mm	45 ± 7	46 ± 6	42 ± 7
LVEF, %	55 ± 7	54 ± 7	56 ± 6
Ablation burden (applications)	67 ± 23	69 ± 25	60 ± 18
Arterial hypertension, n (%)	42 (81 %)	30 (78.9 %)	12 (85.7 %)
Diabetes mellitus, n (%)	6 (12 %)	4 (10.5 %)	2 (14.3 %)
Prior stroke/TIA, n (%)	3 (6 %)	2 (5.3 %)	1 (7.1 %)
Coronary artery disease, n (%)	8 (15 %)	8 (21.1 %)	0 (0 %)
Chronic kidney disease, n (%)	0 (0 %)	0 (0 %)	0 (0 %)
Heart failure, n (%)	7 (13 %)	7 (18.4 %)	0 (0 %)
Dyslipidemia, n (%)	35 (67 %)	26 (68.4 %)	9 (64.3 %)

AF - atrial fibrillation, TIA - transient ischaemic attack, LVEF - left ventricular ejection fraction
